# ﻿150 years after Ferdinand Morawitz: a survey of megachilid bees (Hymenoptera, Megachilidae) of Dagestan, Russia

**DOI:** 10.3897/zookeys.1217.134704

**Published:** 2024-10-30

**Authors:** Alexander V. Fateryga, Maxim Yu. Proshchalykin

**Affiliations:** 1 T.I. Vyazemsky Karadag Scientific Station – Nature Reserve of RAS – Branch of A.O. Kovalevsky Institute of Biology of the Southern Seas of RAS, Nauki Str. 24, Kurortnoye, 298188 Feodosiya, Russia T.I. Vyazemsky Karadag Scientific Station – Nature Reserve of RAS – Branch of A.O. Kovalevsky Institute of Biology of the Southern Seas of RAS Feodosiya Russia; 2 Federal Scientific Center of the East Asia Terrestrial Biodiversity, Far Eastern Branch of the Russian Academy of Sciences, 100-let Vladivostoku Ave. 159, 690022 Vladivostok, Russia Far Eastern Branch of the Russian Academy of Sciences Vladivostok Russia

**Keywords:** Biodiversity, Caucasus, new record, Palaearctic region

## Abstract

A list of 148 species of megachilid bees from 16 genera and five tribes is reported for the Republic of Dagestan. The list is based on more than 2,500 examined specimens and one reliable literature record. Twelve species are new to Russia: Chelostoma (Chelostoma) emarginatum (Nylander, 1856), C. (Foveosmia) maidli (Benoist, 1935), Hoplitis (Alcidamea) campanularis (Morawitz, 1877), H. (A.) caucasica (Friese, 1920), H. (Anthocopa) perezi (Ferton, 1894), H. (Pentadentosmia) tringa (Warncke, 1991), Osmia (Allosmia) melanura Morawitz, 1871, O. (Helicosmia) breviata Warncke, 1988, O. (Osmia) scheherazade Peters, 1978, O. (Pyrosmia) saxicola Ducke, 1899, Anthidium (Anthidium) taeniatum Latreille, 1809, and Megachile (Chalicodoma) montenegrensis Dours, 1873. Nine other species are new to the North Caucasus, and 46 other species are new to Dagestan. Compared to the first list of the bees of Dagestan published by F. Morawitz 150 years ago, the number of species of Megachilidae known from the republic was increased by five times.

## ﻿Introduction

The Republic of Dagestan is the most southern region of Russia. The northern half of Dagestan is a part of the Caspian Depression while its southern half is a part of the Greater Caucasus, one of the most important biodiversity hotspots in the world. The area of Dagestan is somewhat more than 50,000 km^2^, which is not particularly large but the territory of the republic is elevated from −27 to 4,466 m a.s.l. Therefore, landscapes and habitats are extremely diverse and correspondingly changing from lowland deserts to alpine meadows, with a few forest zones as well. The biodiversity of the Republic of Dagestan is also very high; there are more than 3,500 species of vascular plants and 604 species of vertebrates occurring here, while invertebrates are generally poorly studied (Red Book of the Republic of Dagestan 2020). The Megachilidae is a large family of bees numbering more than 4,000 described species worldwide (Michener 2007; Ascher and Pickering 2024); 220 species are known from Russia (Proshchalykin et al. 2023), while knowledge of megachilid bees of Dagestan is very incomplete.

**Figure 1.**
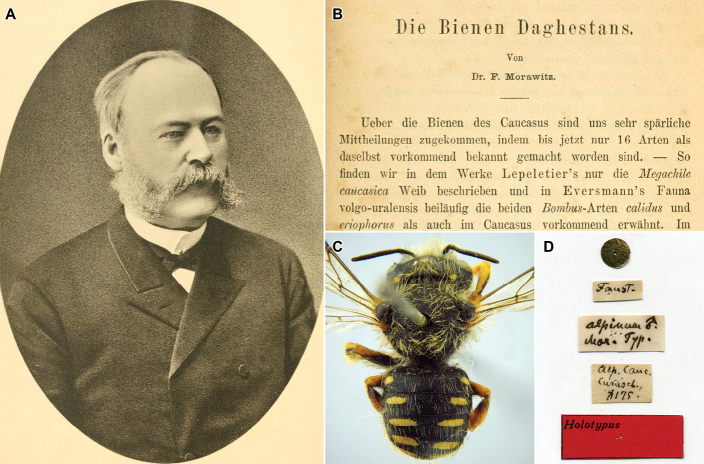
Ferdinand Morawitz and his heritage **A** portrait of F. Morawitz (public domain) **B** beginning of Morawitz’s (1873) paper on the bees of Dagestan (public domain) **C, D** male holotype of *Pseudoanthidiumalpinum* (Morawitz, 1873) described from Dagestan, dorsal view and labels (photographs by Yu. Astafurova).

Ferdinand Morawitz (1827–1896) was one of the leading specialists on the bees (Hymenoptera, Anthophila) at the end of the 19^th^ century (Fig. 1A). He published 64 papers, 44 of them dealing with melittology. In total, Morawitz described five new genera and 725 new species of bees, including 185 species of the family Megachilidae (Pesenko and Astafurova 2003). The vast majority of the species described by him are currently recognized as valid (Schwarz 1980a, 1980b, 1987; Schwarz and Gusenleitner 2002, 2004; Dathe and Proshchalykin 2017; Astafurova and Proshchalykin 2020; Astafurova et al. 2021, 2022). In 1873, Morawitz published the first paper dealing with the bees of Dagestan (Fig. 1B), where he reported 30 species of the family Megachilidae (Morawitz 1873). Six of these species were described as new to science, of which four are currently recognized as valid species (Table 1, Fig. 1C, D). Considering some recently published papers (Fateryga 2017; Fateryga et al. 2019, 2023; Fateryga and Proshchalykin 2020; Litman et al. 2021; Levchenko 2023; Proshchalykin et al. 2023), the number of species of megachilid bees of Dagestan has been increased to 81, which is expected to be still very far from the true number of species occurring in the republic. The purpose of the present contribution is to publish the complete list of all species of megachilid bees known from the Republic of Dagestan to date.

**Table 1.** Species of the family Megachilidae described from Dagestan by Ferdinand Morawitz.

Species name	Sex	Type locality	Current status	Source
*Anthidiumalpinum* Morawitz, 1873	♂	Kurush	Valid, as *Pseudoanthidiumalpinum* (Morawitz, 1873)	Kasparek 2022; Kasparek and Ebmer 2023
*Anthidiumclypeare* Morawitz, 1873	♀	Derbent	Valid, as *Eoanthidiumclypeare* (Morawitz, 1873)	Kasparek 2020, 2022
*Coelioxysconspersa* Morawitz, 1873	♀	Derbent	Junior synonym of *Coelioxyspolycentris* Förster, 1853	Schwarz and Gusenleitner 2003; Fateryga et al. 2019
*Coelioxyspulchella* Morawitz, 1873	♂	Derbent	Junior synonym of *Coelioxyshaemorrhoa* Förster, 1853	Schwarz 2001; Schwarz and Gusenleitner 2003
*Osmianana* Morawitz, 1873	♂	Derbent	Valid	van der Zanden 1991; Warncke 1992
*Osmiaviridana* Morawitz, 1873	♀, ♂	Derbent	Valid	van der Zanden 1991; Warncke 1992

## ﻿Materials and methods

**Figure 2.**
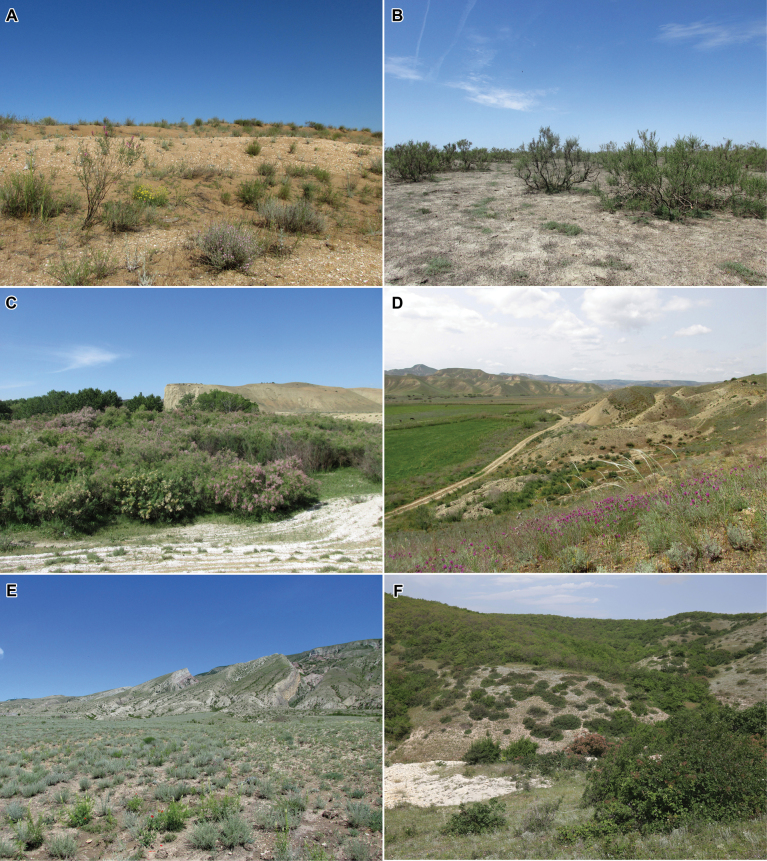
Landscapes of Dagestan **A** coastal dune with flowering *Astragalushyrcanus* Pall., *A.barbidens* Freyn, and *Gelasiabiebersteinii* (Lipsch.) Zaika, Sukhor. & N. Kilian **B** community of *Halostachyscaspica* (M. Bieb.) C.A. Mey. in clay desert **C** flowering *Tamarix* spp. in a river valley **D** steppe slope with flowering *Astragalusbungeanus* Boiss. in foothills **E** clay semi-desert with flowering *Resedaglobulosa* Fisch. & C.A. Mey. in foothills **F** steppe slope with shrubs at oak forest edge on mountain slope.

**Figure 3.**
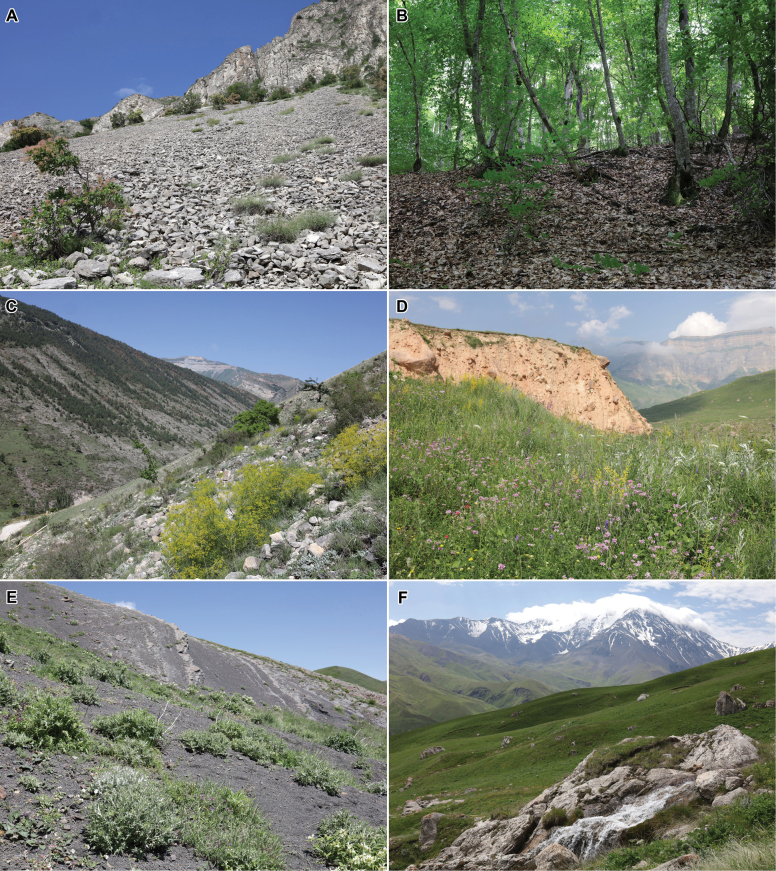
Landscapes of Dagestan **A** limestone scree on mountain slope **B** beech forest on mountain slope **C** limestone mountain slope with flowering *Bilacunariamicrocarpos* (M. Bieb.) Pimenov & V.N. Tikhom. **D** sub-alpine meadow with flowering *Coronillavaria* L., *Galiumverum* L., *Libanotispyrenaica* (L.) Bourg., and other herb species **E** alpine shale scree with flowering *Betonicanivea* Steven on mountain slope **F** alpine meadow.

Several field expeditions were made to various districts of the Republic of Dagestan in 2015–2023, where megachilid bees were collected in all types of landscapes and habitats (Figs 2, 3). Collected specimens are deposited mainly in the collections of the Zoological Institute of the Russian Academy of Sciences, Saint Petersburg, Russia [**ZISP**], the Federal Scientific Center of the East Asia Terrestrial Biodiversity of the Far Eastern Branch of the Russian Academy of Sciences, Vladivostok, Russia [**FSCV**], and the research collections of A.V. Fateryga, Feodosiya, Russia [**CAFK**] and T.V. Levchenko, Moscow, Russia [**CTLM**]. Old material deposited in ZISP was also studied. A total of 2,556 specimens of megachilid bees from Dagestan were examined. Selected specimens were sent to be deposited (some of them temporary) in the Entomological Collection of ETH Zurich, Switzerland [**ETHZ**], Muséum d’Histoire Naturelle de Neuchâtel, Switzerland [**MHNN**], and the research collections of M. Kasparek, Heidelberg, Germany [**CMKH**]. Possible literature sources were also studied but the present work is principally based on material directly examined by the authors and does not include data published online that has not otherwise been validated by experts (e.g., observations reported on iNaturalist). The general distributions of species reported as new to Russia are based on Müller (2024) for the tribe Osmiini, as well as Fateryga et al. (2020), Boustani et al. (2021), and Maharramov et al. (2021) for other taxa.

## ﻿Results

As the result of the study, 2,511 specimens of megachilid bees from Dagestan were identified to 147 species. The remaining 45 specimens represented five species, which identity was unclear. They cannot be identified either without males, as in the case of *Heriades* sp. and Osmia (Pyrosmia) sp., or in the lack of a comprehensive revision of the corresponding group, as in the case of Hoplitis (Hoplitis) spp. and Protosmia (Nanosmia) sp. One more species, Pseudoanthidium (Pseudoanthidium) tenellum (Mocsáry, 1880), was added to the list on the base of a reliable literature record (Litman et al. 2021). A total of 148 species from 16 genera and five tribes were found to occur in Dagestan (Table 2). Full label data of all specimens are represented in Suppl. material 1.

**Table 2.** A list of the megachilid bees of Dagestan (species new to the North Caucasus are indicated with an asterisk; species new to Russia are indicated with two asterisks).

Species name	Literature records	Material examined
**Tribe Lithurgini**		
*Lithurguschrysurus* Fonscolombe, 1834	Fateryga et al. (2019)	43 ♀, 100 ♂
*Lithurguscornutus* (Fabricius, 1787)	Morawitz (1873), as *L.monoceros*, partial misidentification of *L.chrysurus*	4 ♀, 1 ♂
*Lithurgustibialis* Morawitz, 1875	Fateryga et al. (2019)	2 ♀, 1 ♂
**Tribe Osmiini**		
Chelostoma (Chelostoma) emarginatum (Nylander, 1856)**	–	1 ♀, 2 ♂
Chelostoma (Chelostoma) florisomne (Linnaeus, 1758)	–	2 ♀
Chelostoma (Foveosmia) campanularum (Kirby, 1802)	Fateryga et al. (2019)	1 ♀, 9 ♂
Chelostoma (Foveosmia) distinctum (Stöckhert, 1929)	Fateryga et al. (2019)	35 ♀, 24 ♂
Chelostoma (Foveosmia) foveolatum (Morawitz, 1868)	–	3 ♀, 13 ♂
Chelostoma (Foveosmia) maidli (Benoist, 1935)**	–	1 ♂
Chelostoma (Gyrodromella) rapunculi (Lepeletier de Saint-Fargeau, 1841)	–	7 ♀, 34 ♂
Heriades (Heriades) crenulata Nylander, 1856	–	7 ♀, 38 ♂
Heriades (Heriades) rubicola Pérez, 1890	–	59 ♀, 19 ♂
Heriades (Heriades) truncorum (Linnaeus, 1758)	–	8 ♀, 13 ♂
Hoplitis (Alcidamea) acuticornis (Dufour & Perris, 1840)	–	10 ♀, 1 ♂
Hoplitis (Alcidamea) campanularis (Morawitz, 1877)**	–	1 ♀, 3 ♂
Hoplitis (Alcidamea) caucasica (Friese, 1920)**	–	2 ♀
Hoplitis (Alcidamea) curvipes (Morawitz, 1871)	Fateryga and Proshchalykin (2020); Ivanov et al. (2023)	1 ♀, 5 ♀
Hoplitis (Alcidamea) leucomelana (Kirby, 1802)	Morawitz (1873), as *Osmiaparvula*	26 ♀, 46 ♂
Hoplitis (Alcidamea) ozbeki Tkalců, 2000	Proshchalykin et al. (2023)	1 ♀, 1 ♂
Hoplitis (Alcidamea) praestans (Morawitz, 1893)*	–	2 ♀, 4 ♂
Hoplitis (Alcidamea) scita (Eversmann, 1852)*	–	2 ♀, 5 ♂
Hoplitis (Alcidamea) tridentata (Dufour & Perris, 1840)	–	16 ♀, 11 ♂
Hoplitis (Anthocopa) caucasicola Müller, 2012	–	1 ♀
Hoplitis (Anthocopa) jakovlevi (Radoszkowski, 1874)	Fateryga et al. (2019)	3 ♀, 1 ♂
Hoplitis (Anthocopa) mocsaryi (Friese, 1895)	Levchenko (2023)	1 ♀, 1 ♂
Hoplitis (Anthocopa) papaveris (Latreille, 1799)	–	6 ♀, 1 ♂
Hoplitis (Anthocopa) perezi (Ferton, 1894)**	–	1 ♀, 2 ♂
Hoplitis (Hoplitis) adunca (Panzer, 1798)	Morawitz (1873), misidentification of *H.manicata*	50 ♀, 43 ♂
Hoplitis (Hoplitis) anthocopoides (Schenck, 1853)	–	1 ♀, 1 ♂
Hoplitis (Hoplitis) astragali Fateryga, Müller & Proshchalykin, 2023	Fateryga et al. (2023)	46 ♀, 42 ♂
Hoplitis (Hoplitis) dagestanica Fateryga, Müller & Proshchalykin, 2023	Fateryga et al. (2023)	6 ♀, 31 ♂
Hoplitis (Hoplitis) linguaria (Morawitz, 1875)	Proshchalykin et al. (2023)	12 ♀, 4 ♂
Hoplitis (Hoplitis) manicata Morice, 1901	Fateryga et al. (2019)	11 ♀, 17 ♂
Hoplitis (Pentadentosmia) tringa (Warncke, 1991)**	–	2 ♀
Osmia (Allosmia) melanura Morawitz, 1871**	–	10 ♀, 2 ♂
Osmia (Allosmia) rufohirta Latreille, 1811	–	55 ♀, 5 ♂
Osmia (Erythrosmia) andrenoides Spinola, 1808	Fateryga (2017)	12 ♀, 5 ♂
Osmia (Helicosmia) aurulenta (Panzer, 1799)	–	7 ♀, 2 ♂
Osmia (Helicosmia) breviata Warncke, 1988**	–	1 ♀
Osmia (Helicosmia) caerulescens (Linnaeus, 1758)	–	29 ♀, 5 ♂
Osmia (Helicosmia) cinerea Warncke, 1988	Fateryga and Proshchalykin (2020)	3 ♀
Osmia (Helicosmia) dimidiata Morawitz, 1870	–	2 ♀, 5 ♂
Osmia (Helicosmia) leaiana (Kirby, 1802)	–	2 ♀
Osmia (Helicosmia) melanogaster Spinola, 1808	Morawitz (1873), as *O.aterrima*	20 ♀, 9 ♂
Osmia (Helicosmia) niveata (Fabricius, 1804)	Morawitz (1873), as *O.fulviventris*, misidentification of *O.melanogaster*	26 ♀, 2 ♂
Osmia (Helicosmia) signata Erichson, 1835	Morawitz (1873), as *O.melanogastra*	9 ♀, 8 ♂
Osmia (Hoplosmia) bidentata Morawitz, 1875	Fateryga et al. (2019)	5 ♀, 10 ♂
Osmia (Hoplosmia) ligurica Morawitz, 1868	Fateryga and Proshchalykin (2020)	1 ♀
Osmia (Hoplosmia) scutellaris Morawitz, 1868	Morawitz (1873)	3 ♀, 1 ♂
Osmia (Hoplosmia) spinulosa (Kirby, 1802)	Morawitz (1873)	1 ♀, 2 ♂
Osmia (Metallinella) brevicornis (Fabricius, 1798)	Morawitz (1873), as *O.panzeri*	27 ♀, 9 ♂
Osmia (Osmia) apicata Smith, 1853	Fateryga and Proshchalykin (2020)	8 ♀, 5 ♂
Osmia (Osmia) bicornis (Linnaeus, 1758)	Morawitz (1873)	12 ♀
Osmia (Osmia) cornuta (Latreille, 1805)	–	1 ♀, 2 ♂
Osmia (Osmia) mustelina Gerstäcker, 1869	Fateryga and Proshchalykin (2020)	2 ♀
Osmia (Osmia) scheherazade Peters, 1978**	–	1 ♀
Osmia (Pyrosmia) cephalotes Morawitz, 1870	–	35 ♀, 12 ♂
Osmia (Pyrosmia) cyanoxantha Pérez, 1879	Fateryga and Proshchalykin (2020)	1 ♀
Osmia (Pyrosmia) hellados van der Zanden, 1984*	–	4 ♀, 4 ♂
Osmia (Pyrosmia) nana Morawitz, 1873	Morawitz (1873)	1 ♂
Osmia (Pyrosmia) saxicola Ducke, 1899**		1 ♀
Osmia (Pyrosmia) versicolor Latreille, 1811	Fateryga and Proshchalykin (2020)	10 ♀, 6 ♂
Osmia (Pyrosmia) viridana Morawitz, 1873	Morawitz (1873)	35 ♀, 3 ♂
Osmia (Tergosmia) tergestensis Ducke, 1897	–	6 ♀, 9 ♂
Protosmia (Protosmia) glutinosa (Giraud, 1871)	Fateryga and Proshchalykin (2020)	4 ♀
Protosmia (Protosmia) tiflensis (Morawitz, 1876)	Fateryga and Proshchalykin (2020)	7 ♀
**Tribe Anthidiini**		
Anthidiellum (Anthidiellum) strigatum (Panzer, 1805)	Morawitz (1873)	20 ♀, 32 ♂
Anthidiellum (Anthidiellum) troodicum Mavromoustakis, 1949	Proshchalykin et al. (2023)	1 ♀, 1 ♂
Anthidium (Anthidium) cingulatum Latreille, 1809	Fateryga et al. (2019)	11 ♀, 25 ♂
Anthidium (Anthidium) dalmaticum Mocsáry, 1884	Proshchalykin et al. (2023)	2 ♀, 7 ♂
Anthidium (Anthidium) diadema Latreille, 1809	–	1 ♀
Anthidium (Anthidium) florentinum (Fabricius, 1775)	–	48 ♀, 48 ♂
Anthidium (Anthidium) loti Perris, 1852	Fateryga et al. (2019)	7 ♀, 11 ♂
Anthidium (Anthidium) manicatum (Linnaeus, 1758)	–	5 ♀, 2 ♂
Anthidium (Anthidium) melanopygum Friese, 1917	Fateryga et al. (2019), as *A.spiniventre*; Kasparek and Fateryga (2023)	6 ♀, 11 ♂
Anthidium (Anthidium) punctatum Latreille, 1809	Fateryga (2017)	10 ♀, 21 ♂
Anthidium (Anthidium) taeniatum Latreille, 1809**	–	1 ♀, 2 ♂
Anthidium (Proanthidium) oblongatum (Illiger, 1806)	–	11 ♀, 7 ♂
Eoanthidium (Eoanthidium) clypeare (Morawitz, 1873)	Morawitz (1873)	1 ♀
*Icteranthidiumferrugineum* (Fabricius, 1787)	Fateryga et al. (2019)	9 ♀, 9 ♂
*Icteranthidiumgrohmanni* (Spinola, 1838)	Fateryga (2017), misidentification of *I.ferrugineum*; Fateryga et al. (2019)	9 ♀, 4 ♂
Pseudoanthidium (Pseudoanthidium) alpinum (Morawitz, 1873)	Morawitz (1873)	1 ♀, 1 ♂
Pseudoanthidium (Pseudoanthidium) nanum (Mocsáry, 1880)	Morawitz (1873), as *Anthidiumlituratum*; Litman et al. (2021)	6 ♀, 10 ♂
Pseudoanthidium (Pseudoanthidium) stigmaticorne (Dours, 1873)	Litman et al. (2021)	4 ♀, 4 ♂
Pseudoanthidium (Pseudoanthidium) tenellum (Mocsáry, 1880)	Litman et al. (2021)	–
Pseudoanthidium (Royanthidium) melanurum (Klug, 1832)	–	1 ♀, 1 ♂
Pseudoanthidium (Royanthidium) reticulatum (Mocsáry, 1884)	Fateryga et al. (2019)	2 ♂
Stelis (Protostelis) signata (Latreille, 1809)	Fateryga (2017)	1 ♀, 3 ♂
Stelis (Stelidomorpha) nasuta (Latreille, 1809)*	–	3 ♀
Stelis (Stelis) breviuscula (Nylander, 1848)	–	1 ♂
Stelis (Stelis) odontopyga Noskiewicz, 1926*	–	1 ♂
Stelis (Stelis) ornatula (Klug, 1807)	–	5 ♀
Stelis (Stelis) phaeoptera (Kirby, 1802)	Morawitz (1873); Popov (1933), as *S.phaeopterameridionalis*	1 ♀, 1 ♂
Stelis (Stelis) punctulatissima (Kirby, 1802)	–	2 ♂
Stelis (Stelis) scutellaris Morawitz, 1894	–	1 ♀
Trachusa (Archianthidium) pubescens (Morawitz, 1872)	Morawitz (1873)	1 ♀, 10 ♂
Trachusa (Paraanthidium) integra (Eversmann, 1852)	–	2 ♀, 6 ♂
**Tribe Dioxyini**		
*Aglaoapistridentata* (Nylander, 1848)	Fateryga et al. (2019)	4 ♀, 4 ♂
**Tribe Megachilini**		
Coelioxys (Allocoelioxys) acanthura (Illiger, 1806)	Fateryga et al. (2019)	3 ♀, 2 ♂
Coelioxys (Allocoelioxys) afer Lepeletier de Saint-Fargeau, 1841	Morawitz (1873), as *C.coronata*	7 ♀, 16 ♂
Coelioxys (Allocoelioxys) argenteus Lepeletier de Saint-Fargeau, 1841	Morawitz (1873), as both *C.constricta* and *C.argentea*; Fateryga et al. (2019)	3 ♀, 2 ♂
Coelioxys (Allocoelioxys) brevis Eversmann, 1852	Morawitz (1873)	6 ♀, 7 ♂
Coelioxys (Allocoelioxys) caudatus Spinola, 1838	Fateryga et al. (2019)	1 ♀, 2 ♂
Coelioxys (Allocoelioxys) echinatus Förster, 1853	Fateryga and Proshchalykin (2020)	1 ♂
Coelioxys (Allocoelioxys) haemorrhoa Förster, 1853	Morawitz (1873), as *C.pulchella*	3 ♂
Coelioxys (Allocoelioxys) polycentris Förster, 1853	Morawitz (1873), as *C.conspersa*; Fateryga et al. (2019)	11 ♀, 5 ♂
Coelioxys (Coelioxys) quadridentatus (Linnaeus, 1758)*	–	3 ♀, 2 ♂
Coelioxys (Liothyrapis) decipiens (Spinola, 1838)	Fateryga et al. (2019)	1 ♀, 2 ♂
Coelioxys (Melissoctonia) conoideus (Illiger, 1806)	Morawitz (1873), as *C.conoidea*	1 ♀
Coelioxys (Paracoelioxys) elongatus Lepeletier de Saint-Fargeau, 1841	Fateryga and Proshchalykin (2020)	1 ♀
Coelioxys (Paracoelioxys) inermis (Kirby, 1802)	–	1 ♀, 4 ♂
Coelioxys (Paracoelioxys) mandibularis Nylander, 1848*	–	3 ♀, 1 ♂
Coelioxys (Rozeniana) aurolimbatus Förster, 1853	Morawitz (1873), as *C.recurva*	8 ♂
Coelioxys (Rozeniana) rufescens Lepeletier de Saint-Fargeau & Audinet-Serville, 1825	–	7 ♀, 3 ♂
Megachile (Chalicodoma) albocristata Smith, 1853	Morawitz (1873), as *Chalicodomalefebvrei* (misidentified); Fateryga and Proshchalykin (2020)	22 ♀, 8 ♂
Megachile (Chalicodoma) albonotata Radoszkowski, 1886	Fateryga et al. (2019)	14 ♀, 4 ♂
Megachile (Chalicodoma) alborufa Friese, 1911	–	6 ♀, 2 ♂
Megachile (Chalicodoma) montenegrensis Dours, 1873**	–	3 ♂
Megachile (Chalicodoma) parietina (Geoffroy, 1785)	–	10 ♀
Megachile (Creightonella) albisecta (Klug, 1817)	–	21 ♀, 23 ♂
Megachile (Eutricharaea) anatolica Rebmann, 1968*	–	4 ♀, 3 ♂
Megachile (Eutricharaea) apicalis Spinola, 1808	Morawitz (1873), misidentification of *M.versicolor*	12 ♀, 23 ♂
Megachile (Eutricharaea) argentata (Fabricius, 1793)	–	55 ♀, 41 ♂
Megachile (Eutricharaea) burdigalensis Benoist, 1940	Fateryga et al. (2019)	5 ♀, 2 ♂
Megachile (Eutricharaea) deceptoria Pérez, 1890	Fateryga et al. (2019)	24 ♀, 42 ♂
Megachile (Eutricharaea) giraudi Gerstäcker, 1869	Fateryga et al. (2019)	11 ♀, 4 ♂
Megachile (Eutricharaea) leachella Curtis, 1828	Fateryga (2017)	29 ♀, 38 ♂
Megachile (Eutricharaea) leucomalla Gerstäcker, 1869	Fateryga et al. (2019)	4 ♀
Megachile (Eutricharaea) marginata Smith, 1853	Fateryga et al. (2019)	11 ♀, 4 ♂
Megachile (Eutricharaea) rotundata (Fabricius, 1787)	–	19 ♀, 13 ♂
Megachile (Eutricharaea) rubrimana Morawitz, 1893	Fateryga and Proshchalykin (2020)	1 ♀, 1 ♂
Megachile (Eutricharaea) semicircularis auct. nec van der Zanden, 1996	Fateryga et al. (2019)	5 ♀
Megachile (Megachile) centuncularis (Linnaeus, 1758)	–	7 ♀, 10 ♂
Megachile (Megachile) lapponica Thomson, 1872*	–	1 ♀
Megachile (Megachile) ligniseca (Kirby, 1802)	–	1 ♀
Megachile (Megachile) melanopyga Costa, 1863	–	9 ♀, 9 ♂
Megachile (Megachile) octosignata Nylander, 1852	Fateryga and Proshchalykin (2020)	5 ♀
Megachile (Megachile) pilicrus Morawitz, 1877	–	14 ♀, 27 ♂
Megachile (Megachile) versicolor Smith, 1844	–	3 ♀, 9 ♂
Megachile (Pseudomegachile) ericetorum Lepeletier de Saint-Fargeau, 1841	–	18 ♀, 7 ♂
Megachile (Pseudomegachile) flavipes Spinola, 1838	Fateryga et al. (2019)	32 ♀, 11 ♂
Megachile (Pseudomegachile) saussurei Radoszkowski, 1874	Fateryga et al. (2019)	1 ♂
Megachile (Pseudomegachile) tecta Radoszkowski, 1888	Morawitz (1873), as *M.derasa* (misidentified); Fateryga et al. (2019)	16 ♀, 7 ♂
Megachile (Xanthosarus) analis Nylander, 1852	–	1 ♂
Megachile (Xanthosarus) circumcincta (Kirby, 1802)	–	7 ♀, 4 ♂
Megachile (Xanthosarus) lagopoda (Linnaeus, 1761)	–	6 ♀, 11 ♂
Megachile (Xanthosarus) maritima (Kirby, 1802)	Morawitz (1873), misidentification of *M.lagopoda*	5 ♀, 7 ♂
Megachile (Xanthosarus) willughbiella (Kirby, 1802)	–	12 ♀, 9 ♂

Twelve species are reported here from Russia for the first time; their full label data and general distribution are listed below. Besides them, 55 other species are new to Dagestan and nine of them are also reported for the first time from the North Caucasus as a whole: Hoplitis (Alcidamea) praestans (Morawitz, 1893), H. (A.) scita (Eversmann, 1852), Osmia (Pyrosmia) hellados van der Zanden, 1984, Stelis (Stelidomorpha) nasuta (Latreille, 1809), S. (Stelis) odontopyga Noskiewicz, 1926, Coelioxys (Coelioxys) quadridentatus (Linnaeus, 1758), C. (Paracoelioxys) mandibularis Nylander, 1848, Megachile (Eutricharaea) anatolica Rebmann, 1968, and M. (Megachile) lapponica Thomson, 1872 (Table 2). The record of *Hoplitisscita* is especially remarkable because this species was previously known in Russia only from Siberia and the Far East, while its general distribution includes also Kyrgyzstan, Mongolia, and China (Müller 2024).

### ﻿New species records for Russia

#### ﻿ Chelostoma (Chelostoma) emarginatum

Taxon classificationAnimaliaHymenopteraMegachilidae

(Nylander, 1856)

1C0B8E92-1828-5802-BBD2-5C980A208E3F

##### Material examined.

**Russia** • **Dagestan**: Vicinity of Tatil, 42°00'01"N, 48°00'17"E, 4.V.2022, 1 ♂, leg. A. Fateryga [CAFK]; • ibid., 8.V.2022, 1 ♂, leg. A. Fateryga [CAFK]; • ibid., 23.V.2022, 1 ♀, leg. M. Proshchalykin [CAFK].

##### Distribution.

Russia (European part: North Caucasus), Western, Southern, and Eastern Europe, Azerbaijan, Turkey, Iraq, Iran, Turkmenistan.

#### ﻿ Chelostoma (Foveosmia) maidli

Taxon classificationAnimaliaHymenopteraMegachilidae

(Benoist, 1935)

33EB1B5D-FB75-503B-8FDE-F8B55E0F2A87

##### Material examined.

**Russia** • **Dagestan**: Tekipirkent, 41°20'18"N, 47°52'32"E, 29.VI.2023, 1 ♂, leg. A. Fateryga [CAFK].

##### Distribution.

Russia (European part: North Caucasus), Turkey, Syria, Lebanon, Israel.

#### ﻿ Hoplitis (Alcidamea) campanularis

Taxon classificationAnimaliaHymenopteraMegachilidae

(Morawitz, 1877)

B5D80CA3-4ED1-5FE3-AC0C-4E93D06F82D6

##### Material examined.

**Russia** • **Dagestan**: Vicinity of Talgi, 42°52'36"N, 47°26'42"E, 21.V.2022, 1 ♂, leg. A. Fateryga [CAFK]; • ibid., 21.V.2022, 1 ♀, 1 ♂, leg. D. Puzanov [CAFK]; • Dubki, Sulak River, 43°01'50"N, 46°49'29"E, 31.V.2023, 1 ♂, leg. T. Levchenko [CTLM].

##### Distribution.

Russia (European part: North Caucasus), Southern and Eastern Europe, North Africa, Georgia, Turkey, Lebanon, Israel.

#### ﻿ Hoplitis (Alcidamea) caucasica

Taxon classificationAnimaliaHymenopteraMegachilidae

(Friese, 1920)

6B50413F-DC61-55CD-99A3-3C9EC917C0B6

##### Material examined.

**Russia** • **Dagestan**: Tsudakhar, 42°19'43"N, 47°09'51"E, 15.VI.2023, 2 ♀, leg. M. Proshchalykin [CAFK, ETHZ].

##### Distribution.

Russia (European part: North Caucasus), Azerbaijan, Turkey.

#### ﻿ Hoplitis (Anthocopa) perezi

Taxon classificationAnimaliaHymenopteraMegachilidae

(Ferton, 1894)

C22138CC-3E14-5703-A66A-200492265A92

##### Material examined.

**Russia** • **Dagestan**: 7 km SE Gedzhykh, 42°03'52"N, 48°05'57"E, 3.VI.2019, 1 ♀, 1 ♂, leg. M. Proshchalykin, V. Loktionov [FSCV]; • Derbent, railroad to the north from the fortress wall, on *Convolvulusarvensis*, 4.VII.2022, 1 ♂, leg. T. Levchenko [CTLM].

##### Distribution.

Russia (European part: North Caucasus), Western, Southern, and Eastern Europe, North Africa, Armenia, Azerbaijan, Turkey, Israel, Iran, Afghanistan, Turkmenistan, Tajikistan, Uzbekistan, Kyrgyzstan, Kazakhstan.

#### ﻿ Hoplitis (Pentadentosmia) tringa

Taxon classificationAnimaliaHymenopteraMegachilidae

(Warncke, 1991)

69AFD11A-58BE-57CD-8459-7E4E5310D874

##### Material examined.

**Russia** • **Dagestan**: Tsudakhar, 42°19'43"N, 47°09'51"E, 15.VI.2023, 2 ♀, leg. M. Proshchalykin [CAFK, ETHZ].

##### Distribution.

Russia (European part: North Caucasus), Azerbaijan, Turkey, Iran.

#### ﻿ Osmia (Allosmia) melanura

Taxon classificationAnimaliaHymenopteraMegachilidae

Morawitz, 1871

8F66A6A8-622C-5FDC-A38C-68D79BB9C059

##### Material examined.

**Russia** • **Dagestan**: Gelinbatan, 41°56'30"N, 48°10'41"E, 5.V.2022, 8 ♀, 2 ♂, leg. A. Fateryga [CAFK]; • ibid., on *Onobrychismajorovii*, 5.V.2022, 1 ♀, leg. A. Fateryga [CAFK]; • Kamyshchay River valley, 41°54'33"N, 48°13'47"E, on *Astragalusbungeanus*, 5.V.2022, 1 ♀, leg. A. Fateryga [CAFK].

##### Distribution.

Russia (European part: North Caucasus), Southern and Eastern Europe, Armenia, Azerbaijan, Turkey.

#### ﻿ Osmia (Helicosmia) breviata

Taxon classificationAnimaliaHymenopteraMegachilidae

Warncke, 1988

5D42E153-48BF-552A-94BF-A518A6860985

##### Material examined.

**Russia** • **Dagestan**: Khotoch, 42°24'52"N, 46°57'10"E, 17.VI.2023, 1 ♀, leg. M. Proshchalykin [ETHZ].

##### Distribution.

Russia (European part: North Caucasus), Southern Europe, Turkey, Lebanon, Israel, Iran.

#### ﻿ Osmia (Osmia) scheherazade

Taxon classificationAnimaliaHymenopteraMegachilidae

Peters, 1978

675D78C2-21DC-5354-9691-CE749476B355

##### Material examined.

**Russia** • **Dagestan**: 5 km NNW Chirag, 41°52'47"N, 47°23'25"E, 25.VI.2023, 1 ♀, leg. M. Proshchalykin [CAFK].

##### Distribution.

Russia (European part: North Caucasus), Turkey, Iran.

#### ﻿ Osmia (Pyrosmia) saxicola

Taxon classificationAnimaliaHymenopteraMegachilidae

Ducke, 1899

B0C71B33-C469-5568-BB84-C442F057E5AA

##### Material examined.

**Russia** • **Dagestan**: Tsudakhar, 42°19'43"N, 47°09'51"E, 28–29.V.2022, 1 ♀, leg. M. Proshchalykin [CAFK].

##### Distribution.

Russia (European part: North Caucasus), Southern and Eastern Europe, Turkey, Cyprus, Syria, Jordan, Lebanon, Israel, Iraq, Iran, Tajikistan.

#### ﻿ Anthidium (Anthidium) taeniatum

Taxon classificationAnimaliaHymenopteraMegachilidae

Latreille, 1809

EE370D96-1DC7-53DE-853A-23A52B9811B0

##### Material examined.

**Russia** • **Dagestan**: Belidzhi, hot spring, 41°54'2"N, 48°26'14"E, on *Lotuscorniculatus*, 10.VI.2023, 1 ♀, 2 ♂, leg. T. Levchenko [CTLM].

##### Distribution.

Russia (European part: North Caucasus), Western, Southern, and Eastern Europe, Azerbaijan, Turkey, Lebanon, Israel, Iran, Turkmenistan.

#### ﻿ Megachile (Chalicodoma) montenegrensis

Taxon classificationAnimaliaHymenopteraMegachilidae

Dours, 1873

02EE1019-AAAB-5795-98DD-18CA3189D68A

##### Material examined.

**Russia** • **Dagestan**: Vicinity of Gubden, 42°34'23"N, 47°33'01"E, 2.VI.2022, 1 ♂, leg. A. Fateryga [MHNN]; • ibid., 3.VI.2022, 2 ♂, leg. A. Fateryga [CAFK].

##### Distribution.

Russia (European part: North Caucasus), Southern and Eastern Europe, North Africa, Armenia, Azerbaijan, Turkey, Syria, Lebanon, Israel, Iran, Afghanistan, Tajikistan, Uzbekistan.

## ﻿Discussion

The first paper on the bees of the Republic of Dagestan was published 150 years ago by Morawitz (1873) and it contained 30 species of the family Megachilidae, including a species later synonymized (*Coelioxysconstrictus* Förster, 1853 with *C.argenteus* Lepeletier de Saint-Fargeau, 1841); some other species were misidentified (Table 2). Recently published papers (Fateryga 2017; Fateryga et al. 2019, 2023; Fateryga and Proshchalykin 2020; Litman et al. 2021; Levchenko 2023; Proshchalykin et al. 2023) added 52 species, including two species described as new to science (Fateryga et al. 2023). By this way, the total number of species of megachilid bees of Dagestan has increased to 81. The present contribution reports a total of 148 species of megachilid bees known from Dagestan. Compared to the first list published by Morawitz (1873), the number of species known from the republic was increased by five times.

Thirty-two species recorded in Dagestan are widespread in the whole Palaearctic region: *Lithurguscornutus*, *Chelostomafoveolatum*, *C.rapunculi*, *Heriadestruncorum*, *Hoplitisleucomelana*, *H.tridentata*, *Osmialeaiana*, *Anthidiellumstrigatum*, *Anthidiumflorentinum*, *A.punctatum*, *Stelisornatula*, *Coelioxysafer*, *C.brevis*, *C.conoideus*, *C.elongatus*, *C.haemorrhoa*, *C.inermis*, *C.mandibularis*, *C.quadridentatus*, *C.rufescens*, *Megachileanalis*, *M.centuncularis*, *M.circumcincta*, *M.ericetorum*, *M.lagopoda*, *M.lapponica*, *M.ligniseca*, *M.maritima*, *M.melanopyga*, *M.rotundata*, *M.versicolor*, and *M.willughbiella*.

Fifteen species are widespread in West Palaearctic: *Lithurguschrysurus*, *Chelostomacampanularum*, *C.florisomne*, *Heriadescrenulata*, *Hoplitisanthocopoides*, *H.curvipes*, *H.manicata*, *Osmiamelanura*, *O.tergestensis*, *Pseudoanthidiumalpinum*, *Stelisodontopyga*, *Trachusaintegra*, *Megachileburdigalensis*, *M.leachella*, and *M.octosignata*.

Fifty species are distributed in Europe to Caucasus and Central Asia: *Lithurgustibialis*, *Heriadesrubicola*, *Hoplitisacuticornis*, *H.adunca*, *H.jakovlevi*, *H.perezi*, *H.praestans*, *H.papaveris*, *Osmiabicornis*, *O.brevicornis*, *O.caerulescens*, *O.cephalotes*, *O.cornuta*, *O.dimidiata*, *O.spinulosa*, *O.viridana*, *Anthidiumcingulatum*, *A.diadema*, *A.loti*, *A.manicatum*, *A.oblongatum*, *A.taeniatum*, *Icteranthidiumferrugineum*, *I.grohmanni*, *Pseudoanthidiumtenellum*, *Stelisbreviuscula*, *S.nasuta*, *S.phaeoptera*, *S.punctulatissima*, *S.scutellaris*, *S.signata*, *Aglaoapistridentata*, *Coelioxysacanthura*, *C.aurolimbatus*, *C.argenteus*, *C.caudatus*, *C.decipiens*, *Megachilealbisecta*, *M.apicalis*, *M.argentata*, *M.deceptoria*, *M.flavipes*, *M.giraudi*, *M.marginata*, *M.montenegrensis*, *M.parietina*, *M.pilicrus*, *M.rubrimana*, *M.saussurei*, and *M.tecta*.

Nearly a third of the megachilid fauna of Dagestan is formed by species with smaller ranges or endemic distributions. Forty species are distributed from Southern Europe to the Caucasus, or from the Mediterranean to the Middle East and the Caucasus (some species also occur in Iran, north-western Turkmenistan Afghanistan, and Pakistan): *Chelostomadistinctum*, *C.emarginatum*, *C.maidli*, *Hoplitiscampanularis*, *H.mocsaryi*, *Osmiaandrenoides*, *O.apicata*, *O.aurulenta*, *O.bidentata*, *O.breviata*, *O.cyanoxantha*, *O.hellados*, *O.ligurica*, *O.melanogaster*, *O.mustelina*, *O.nana*, *O.niveata*, *O.rufohirta*, *O.saxicola*, *O.scutellaris*, *O.signata*, *O.versicolor*, *Protosmiaglutinosa*, *P.tiflensis*, *Anthidiellumtroodicum*, *Anthidiumdalmaticum*, *A.melanopygum*, *Eoanthidiumclypeare*, *Pseudoanthidiumnanum*, *P.melanurum*, *P.reticulatum*, *P.stigmaticorne*, *Trachusapubescens*, *Coelioxysechinatus*, *C.polycentris*, *Megachilealbocristata*, *M.albonotata*, *M.anatolica*, *M.leucomalla*, and *M.semicircularis.* One species has a remarkably disjunctive distribution in the Caucasus and eastern Central Asia to the Far East: *Hoplitisscita*. Ten species are endemic or subendemic to the Caucasus and Turkey (some of them also occur in Iran or north-western Turkmenistan): *Hoplitisastragali*, *H.caucasica*, *H.caucasicola*, *H.dagestanica*, *H.linguaria*, *H.ozbeki*, *H.tringa*, *Osmiacinerea*, *O.scheherazade*, and *Megachilealborufa*; and one of them (*H.dagestanica*) is known only from Dagestan.

Thus, the fauna of Dagestan is very diverse and consists of species with wide Palaearctic or Western Palaearctic ranges, as well as elements of Mediterranean, European, Central Asian faunas and a relatively small number of endemic species.

According to the studied material, *Lithurguschrysurus*, *Anthidiumflorentinum*, *Megachileargentata*, *Hoplitisadunca*, *H.astragali*, *Heriadesrubicola*, *Hoplitisleucomelana*, *Megachileleachella*, *M.deceptoria*, and *Osmiarufohirta* are the most common species of megachilid bees in Dagestan, with ≥ 60 collected specimens. At the same time, 20 species are known by one specimen each (Table 2). Only 39 species of megachilid bees were recorded in the northern half of Dagestan (a part of the Caspian Depression) and just three of them (*Pseudoanthidiumtenellum*, *Coelioxysdecipiens*, and *Megachilesaussurei*) were recorded only there. In the southern half of the republic (a part of the Greater Caucasus), 145 species were recorded. Among the four major landscape zones of this territory, the richest megachilid-bee fauna was revealed in the belt of foothills (109 species). The belt of so-called Intramountain Dagestan numbered 70 species of megachilid bees, 65 species were revealed in the coastal lowland, and 49 in the high mountain belt.

**Figure 4.**
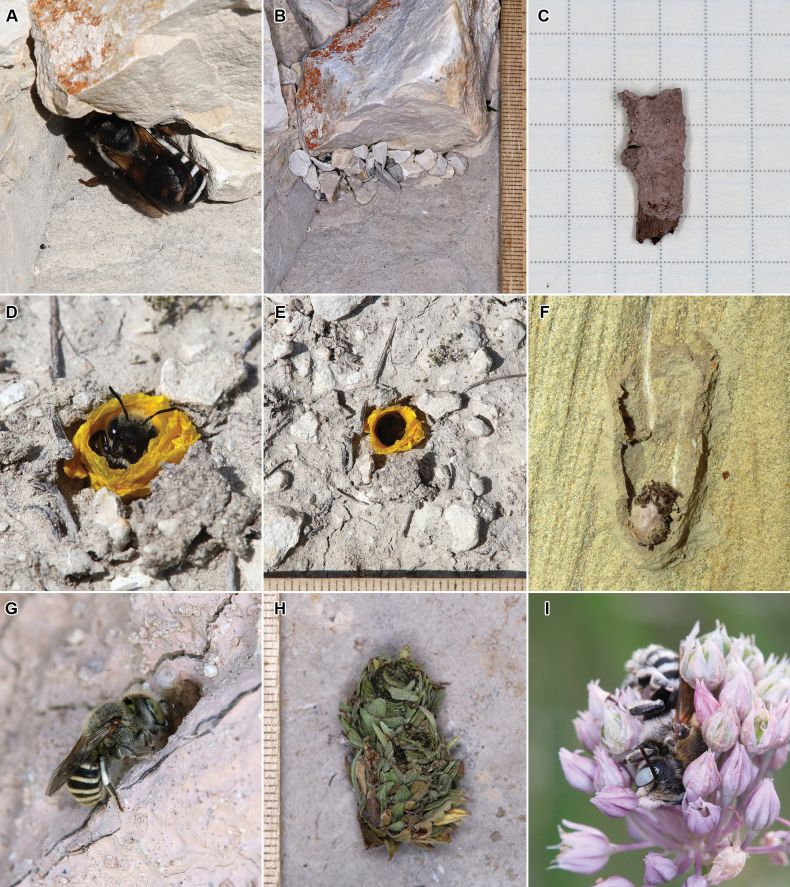
Biology of megachilid bees from Dagestan **A** female of *Megachilealbocristata* Smith, 1853 at her nest entrance **B** same nest sealed with pebbles **C** nest cell of *Megachileflavipes* Spinola, 1838 extracted from the substrate **D** female of *Hoplitismocsaryi* (Friese, 1895) at her nest entrance **E** same nest entrance from above **F** dissected old nest cell of *Sceliphron* sp. with a cell of *Osmiacornuta* (Latreille, 1805) containing a cocoon **G** female of *Hoplitisastragali* Fateryga, Müller & Proshchalykin, 2023 closing her nest with a plug of mud **H** nest of *Hoplitiscurvipes* (Morawitz, 1871) extracted from the substrate **I** male of *H.curvipes* sleeping in an inflorescence of *Alliumrotundum* L. s. l.

Twenty-five species of megachilid bees of 148 are kleptoparasitic taxa of the genera *Stelis* Panzer, 1806, *Aglaoapis* Cameron, 1901, and *Coelioxys* Latreille, 1809. The remaining 123 species are nest building. In the curse of our fieldwork in Dagestan, we recorded nests of nine species of megachilid bees. Nests of *Hoplitisadunca*, *Osmiacaerulescens*, and *O.dimidiata* were recorded in trap nests made of reed stems. Biology of all three species was well studied previously (summarised by Müller 2024). The nests of *O.caerulescens* were especially numerous. A nest of *Megachilealbocristata* was found between stones (Fig. 4A). The nest was subsequently sealed by the female bee with pebbles fastened with leaf pulp (Fig. 4B). A nest of *Megachileflavipes* was found in an abandoned nest hole of *Anthophora* sp. (Hymenoptera, Apidae) on a clay cliff. The nest consisted of two cylindrical mud cells (Fig. 4C). A nest of *Hoplitismocsaryi* was found in the ground, on horizontal surface. The nest entrance was lined with fragments of petals of *Linumtauricum* Willd. (Fig. 4D, E). This bee species is well known to use flax petals (Ivanov and Filatov 2008; Levchenko 2023). Two nests of *Osmiacornuta* were revealed in abandoned nest cells of *Sceliphron* sp. (Hymenoptera, Sphecidae). This bee species is well known to use various pre-existing cavities for nesting (summarised by Müller 2024). Six nests of *Hoplitisastragali* were revealed on a clay cliff (Fig. 4G); females of this species excavated burrows by themselves and used mud for nest construction. The nests were described in detail by Fateryga et al. (2023). The most remarkable nest found in Dagestan was that of *Hoplitiscurvipes*. It consisted of two cells placed side by side under a stone; the cells were constructed from leaf fragments, which were imbricately arranged, forming a cone-like structure; each leaf fragment consisted of a basal part that was masticated to leaf pulp and an apical part that protruded freely from the cell wall (Fig. 4H). The nest of this species was described in detail by Ivanov et al. (2023). Males of *Hoplitiscurvipes* were recorded sleeping in inflorescences of *Alliumrotundum* L. s. l. (Fig. 4I).

The megachilid-bee fauna of Dagestan is rich. Almost 2/3 of all species known from Russia (232 according to Proshchalykin et al. 2023 and present data) occur in Dagestan, while the area of Dagestan is about 0.3% of the area of Russia. The megachilid-bee fauna of Dagestan is less diverse but still comparable to that of neighbouring Azerbaijan, which has 175 species of megachilid bees (Maharramov et al. 2023; Fateryga et al. 2023), while the area of Azerbaijan is more than one and a half times more than that of Dagestan. A comparison of the list of megachilid bees of Azerbaijan (compiled from Fateryga et al. 2020; Proshchalykin and Maharramov 2020; Maharramov et al. 2021, 2023; Fateryga et al. 2023) with that of Dagestan revealed that 109 species (51%) occur in both territories. Our results also show that the knowledge of the family Megachilidae of Dagestan is still incomplete. Despite the reached progress, several species remained unidentified, and this problem may be solved only in the curse of special taxonomic investigations of particular subgenera and groups of species. Biology of many species occurring in Dagestan is unknown and should be also studied during further research.

## Supplementary Material

XML Treatment for Chelostoma (Chelostoma) emarginatum

XML Treatment for Chelostoma (Foveosmia) maidli

XML Treatment for Hoplitis (Alcidamea) campanularis

XML Treatment for Hoplitis (Alcidamea) caucasica

XML Treatment for Hoplitis (Anthocopa) perezi

XML Treatment for Hoplitis (Pentadentosmia) tringa

XML Treatment for Osmia (Allosmia) melanura

XML Treatment for Osmia (Helicosmia) breviata

XML Treatment for Osmia (Osmia) scheherazade

XML Treatment for Osmia (Pyrosmia) saxicola

XML Treatment for Anthidium (Anthidium) taeniatum

XML Treatment for Megachile (Chalicodoma) montenegrensis

## ﻿Additional information

### Author ORCIDs

Alexander V. Fateryga https://orcid.org/0000-0002-5346-3477

Maxim Yu. Proshchalykin https://orcid.org/0000-0001-7870-8226

### Data availability

All of the data that support the findings of this study are available in the main text or Supplementary Information.

## Supplementary materials

Supplementary material 1 List of specimens examined Data type xls Explanation note List of all 2556 examined specimens of the megachilid bees from Dagestan. https://binary.pensoft.net/file/1164398This dataset is made available under the Open Database License (http://opendatacommons.org/licenses/odbl/1.0/). The Open Database License (ODbL) is a license agreement intended to allow users to freely share, modify, and use this Dataset while maintaining this same freedom for others, provided that the original source and author(s) are credited.Maxim Yu. Proshchalykin, Alexander V. Fateryga
